# Hybrid Analytical and Data-Driven Modeling for Feed-Forward Robot Control †

**DOI:** 10.3390/s17020311

**Published:** 2017-02-08

**Authors:** René Felix Reinhart, Zeeshan Shareef, Jochen Jakob Steil

**Affiliations:** 1 Fraunhofer Research Institute for Mechatronic Systems Design (IEM), Zukunftsmeile 1, 33102 Paderborn, Germany; felix.reinhart@iem.fraunhofer.de; 2 Institute for Robotics and Process Control (IRP), Technische Universität Braunschweig, Mühlenpfordstraße 23, 38106 Braunschweig, Germany; jsteil@rob.cs.tu-bs.de

**Keywords:** machine learning, data-driven modeling, mechanical modeling, error models, inverse kinematics, inverse dynamics, learning feed-forward control, soft robot

## Abstract

Feed-forward model-based control relies on models of the controlled plant, e.g., in robotics on accurate knowledge of manipulator kinematics or dynamics. However, mechanical and analytical models do not capture all aspects of a plant’s intrinsic properties and there remain unmodeled dynamics due to varying parameters, unmodeled friction or soft materials. In this context, machine learning is an alternative suitable technique to extract non-linear plant models from data. However, fully data-based models suffer from inaccuracies as well and are inefficient if they include learning of well known analytical models. This paper thus argues that feed-forward control based on hybrid models comprising an analytical model and a learned error model can significantly improve modeling accuracy. Hybrid modeling here serves the purpose to combine the best of the two modeling worlds. The hybrid modeling methodology is described and the approach is demonstrated for two typical problems in robotics, i.e., inverse kinematics control and computed torque control. The former is performed for a redundant soft robot and the latter for a rigid industrial robot with redundant degrees of freedom, where a complete analytical model is not available for any of the platforms.

## 1. Introduction

Progress in intelligent automation systems and towards novel products often requires improved models for control. However, the systems are often too complex to derive their action-effect relationships in simple models and to fully capture their dynamics. Plant parameters may be time varying or unknown, or the models demand a high computational effort, which renders model-based control infeasible. Additionally, for feed-forward (or open-loop) control typically an inverse model is required, which is even more difficult to derive for instance in the presence of redundancies in the plant. Therefore feed-forward control is often implemented by local inversion of a forward model. Analytical modeling refers in this context to the availability of a physics-based, algebraic description of the plant, which often must rely on approximations and sometimes simplifications. For such analytical models, parameter identification based on example data and regression methods is often required in the modeling process. In robotics, typically exciting oscillatory movements are executed for recording respective data [1] and optimal excitation for model identification is researched in [2].

On the other hand, machine learning is a suitable approach to extract non-linear plant models from data. Regression techniques have previously been applied to learn inverse models for feed-forward control in robotics, e.g., [3,4,5], where acquisition of input-output pairs for supervised learning is achieved in [4,5] by relying on low-gain feedback control. Explorative learning of inverse kinematics has also been addressed in much generality in [6,7]. To establish data-driven modeling in inverse dynamics, the joints’ information $\left(q,\dot{q},\ddot{q}\right)$ can be mapped directly to joint torques [8,9,10,11] by solving a regression problem. One difficulty of these approaches is that supervised training data needs to be collected which comprises pairs of desired effects and appropriate actions. This data has to represent the highly non-linear mapping in a high-dimensional state space, which becomes increasingly difficult with an increasing number of joints. Regardless of the concrete application and the type of machine learning approach employed, data which characterizes the plant has to be collected.

This paper argues that it can be more effective to design a hybrid model based on the combination of an approximate analytical model plus a learned, data-driven error model than to derive a purely analytic or data-driven model. A hybrid model can potentially capture more complex characteristics like friction, material properties, etc., while not dispensing with the advantage of the interpretability and expressiveness of an analytical model.

Hybrid modeling has appeared in the literature relatively scarcely so far. Some recent examples are primarily from the robotics domain but not restricted to it. For instance in [12], a learned classifier is combined with analytical models to detect the time of optimal dough quality for an industrial dough kneading machine. In robotics, hybrid approaches for modeling of inverse kinematics and dynamics include [10,13,14,15,16,17]. Reinhart et al. [15] developed a hybrid kinematics model for a soft robot that is described in detail in Section 3 below. Nguyen-Tuong et al. [10] showed that available prior knowledge of a robot’s dynamic model can entail valuable information for model learning that may result in faster learning speed, higher accuracy and better generalization. Terry Taewoong Um et al. [13] proposed a hybrid modeling method called Independent Joint Learning (IJL) that will also be employed in this paper. It combines a classical inverse dynamics model of a robotic manipulator using the recursive Newton-Euler analytical method with a learned error model. The latter is trained on the error between the torques obtained from the real robot and those obtained using the analytical inverse dynamics model. Terry Taewoong Um et al. [13] applied this approach to the PUMA 560 arm in simulation, while in [14] this approach has been successfully applied to improve the existing ID model of a real three Degrees of Freedom (3-DoF) arm for the COmpliant huMANoid (COMAN) robot. Very recent work [18] has discussed the online learning of inverse dynamics by adding an adaptive offset term to the model that ensures good tracking.

The hybrid modeling methodology has been addressed only implicitly in these works and is typically hidden in the algorithmic approach. For instance in [10], the analytical model is treated as being in service of the learning and for simplifying learning, whereas the current paper argues that essentially both the analytical and the data-driven parts play their well-defined roles in a more coherent overall hybrid modeling approach. To make this explicit, this paper describes the methodology in some generality and points out common principles, while illustrating these through examples from robotics.

After discussing the methodology, the paper demonstrates the benefits of the hybrid approach in feed-forward control in two typical control contexts in robotics, namely for inverse velocity kinematics control and for inverse dynamics torque control. In both cases, the derived hybrid forward models comprise an analytical model and a learned error model and can significantly improve accuracy of positioning and tracking tasks. It is systematically pointed out that hybrid modeling in this form has certain advantages also as compared to learning the entire model purely data driven: (a) Restricting learning to the error of the mechanical model reduces the complexity of the to-be-learned model; (b) Non-linearities captured by the mechanical model reduce the training data demand for learning the error model; (c) The feed-forward control law is based on the combination of the inverse analytical model, which mostly features robust extrapolation and high expressive power, and a black-box error model, which can be restricted to a certain degree such that unintended extrapolation artifacts are mitigated.

**Figure 1:**
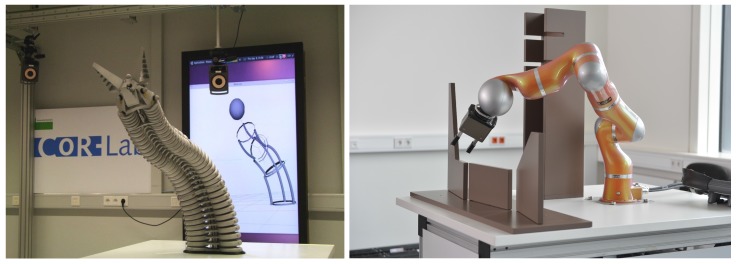
**Left**: The Bionic Handling Assistant (BHA) is a pneumatically actuated, soft robot trunk manufactured by Festo (figure reproduced from [19]). **Right**: The rigid industrial manipulator KUKA Lightweight Robot (LWR) IV+ features seven degrees of freedom with joint torque sensors.

We demonstrate the proposed methodology for inverse kinematic control of a redundant soft robot trunk and for modeling the inverse dynamics of a rigid industrial manipulator (see Figure 1). For the soft robot, a hybrid model is constructed from approximate constant curvature continuum kinematics [19,20] and an error model which is implemented in form of an efficient neural network. The accuracy of the hybrid model is compared to the continuum kinematics model alone and an entirely learned forward kinematic model. Feed-forward control of the end effector is then achieved by inversion of the hybrid model as in Jacobian-based inverse velocity kinematics for redundant robots. In this case study, we demonstrate that the choice of the learning algorithm and the possibility for inversion of the model are important.

In the example of a rigid industrial manipulator, we utilize the analytical inverse dynamics model of the manipulator that is derived by using the recursive Newton-Euler method, however, based on imprecise data about the mechanism, because the manufacturer does not disclose the precise model. This approximation adds to the inevitable unmodeled dynamics of the manipulator. Again the same neural network learner is used to estimate the error between the torques obtained from the real robot and those obtained using the analytical inverse dynamics model. The paper specifically investigates in this case, which selection of inputs for the data-driven learning leads to the most accurate torque error model, illustrating the problem that the features which are used as input to the learner have to be expressive in order to allow for successful modeling. The results show that a significantly more accurate dynamics model can be obtained for the manipulator through this hybrid modeling as compared to the stand-alone analytical model.

The remainder of the paper is organized as follows. In Section 2, the hybrid modeling methodology is described and discussed in some generality. Section 3 introduces the example case of the kinematic control of the continuum soft robot, while Section 4 tackles the case of inverse dynamics control. Finally, the paper concludes with general implications of the hybrid feed-forward control scheme for improving controller design. This paper extends our results in previous work [15,16] by a more in-depth discussion of properties and related work in hybrid modeling, by reporting additional results, as well as by discussing the results in a larger context.

## 2. Hybrid Modeling

**Figure 2:**
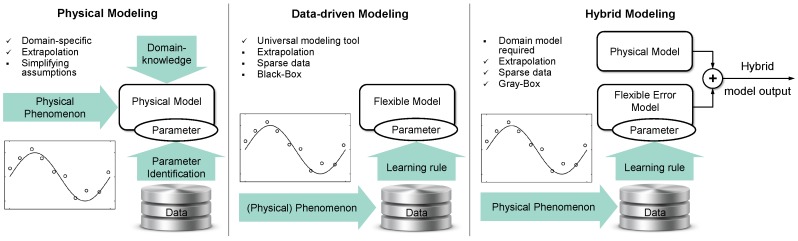
**Left**: Classical, e.g., physical, modeling targets a compact, domain-specific formulation. **Middle**: Data-driven modeling utilizes flexible models that are problem-agnostic and typically employ universal function approximators. **Right**: Hybrid modeling, as it is proposed in this paper, combines an analytical model with a data-driven error model.

Classical, analytical modeling, often derived from the basic laws of physics, targets a compact, domain-specific formulation (see Figure 2 (left)). Physical models extrapolate well by design and therefore are preferred for model-based control approaches. However, there may remain modeling errors, for instance in robotics due to imprecise knowledge of the mass distribution of the manipulator, lack of suitable friction models, backlash, or unmodeled elasticity in the mechanism. There are multiple sources of error that can in general corrupt the modeling. Besides numerical and measurement errors, simplifying assumptions that are necessary to derive a model in the first place can cause significant residual errors. For instance in case of very complex mechanism like humanoid robots, only approximate models are compact and efficient enough to be feasible for control. For instance, in the control of walking often spring loaded inverted pendulum (SLIP) models are used (e.g., [21]). The perturbations that arise from such approximations and simplifications are rejected by means of PID controllers in respective control schemes. An improvement of accuracy in the modeling then directly converts into a reduction of gains for these controllers. This reduction consequently is a major objective and performance criterion for the success of such modeling approaches [14,16,18].

On the contrary, data-driven modeling approaches utilize flexible internal representations that are problem-agnostic and most often are universal function approximators (see Figure 2 (Middle)). However, extrapolation is limited and data can hardly ever exhaust all possible plant configurations resulting in sparse data. Therefore careful selection of the training data, training algorithm, and model complexity is required. In learning of inverse models in the presence of redundancy, additionally the selection of consistent training data that do not include non-convex and thus incompatible redundancy resolutions is an additional issue [6]. Furthermore, the black-box character of many learning techniques complicates understanding and analysis of the properties of the resulting model. A particular problem occurs in robotics where models have to be inverted for control, which calls for specialized learning methods based on suitable internal representations.

In this paper, a hybrid model is defined as a combination of a rigid, inaccurate, but possibly parametrized, analytical model with a learned, data-driven error model (see Figure 2 (right)). It targets to capture the “best of both worlds”, which are briefly described above. The hybrid model requires a domain-specific plant model, e.g., a mechanical model, which is able to extrapolate and capture important physical and possibly non-linear aspects of the modeling task. The data-driven error model then substitutes for model approximations and inaccuracies. The resulting hybrid model has a gray-box character due to the mixture of a domain-specific part and the black-box error model.

The mutual benefits are obvious. If essential aspects and non-linearities of the plant are already captured by the domain-specific model, the error model can have lower model complexity than a fully learned model and thus does not suffer from overfitting from sparse data. On the other hand, a learned model may capture unmodeled dynamics and thereby improve the accuracy over the analytical model alone. While examples are provided in the context of robotics, the following four essential ingredients are needed for a hybrid approach in any domain and the respective deliberations are of rather general nature.

(A) **The analytical model:** In principle, any analytical model of a plant can be used in hybrid modeling. Naturally, the better and more accurate this model is, the easier the remaining learning task. Therefore, while in theory very coarse approximations could be used, it will in practice be important to capture at least some of the main non-linearities and difficulties of the task-relevant mechanism in the analytical part. Here, the relation to the task, usually a specific control objective, is important. For instance, in robotics the analytical inverse dynamics model, which is used for computed torque-control, requires kinematic information, however in an implicit way. The form of this dynamic model is textbook knowledge and no explicit kinematics model is needed. In inverse kinematics velocity control, however, the inverse of the explicit kinematics is needed for control. Then we have different options for modeling, namely forward kinematics modeling plus local inversion or direct inverse kinematics modeling. Both lead to respectively different approaches in hybrid modeling as well, because in the former case the learned error model also has to be inverted. In the latter case different and not so easy to obtain training data is needed due to redundancies of the kinematics and resulting non-convexity of the direct inverse modeling problem [6]. Furthermore, reduced models are used in practice like the already mentioned SLIP model for floating bases. Another example, as discussed in Section 3 below, is approximate continuum kinematics which may be used for soft robots, because no better model is easily available. In these cases, a hybrid model additionally has to deal with the approximation errors.
(B) **The data:** In the hybrid modeling approach, data is always needed to train the error model, but often also for parameter identification of the analytical model. Ideally, the same data can be used and the learning approach can benefit from parameter identification theory. Methods to excite the plant in optimal ways to reflect all relevant dynamical phenomena in the measurable data have been studied in that domain, e.g., [2]. To obtain such data is also a precondition for the learning approach, because nothing can be captured through machine learning that is not present in the data. However, for machine learning the issue of data acquisition is more intricate because the amount and density of the available data may strongly determine the choice of the learning algorithm and its underlying representation model. The issue of overfitting, that is too high-model complexity that fits too few data too well and then leads to poor generalization, is a persistent issue for robots and other physical plants. Overfitting can be mitigated by collecting additional data. However, data collection is expensive if real-world action must be executed to generate a training sample. Thus the “know your data”-principle also applies in the hybrid modeling context as in any data-based method, whereas data acquisition can benefit from well-founded data collection schemes from the domain.
(C) **The learning algorithm:** The choice of the learning algorithm and its underlying representation naturally has an important impact on the overall performance of the approach. In principle, any learner may be used and various approaches have been tried in practice. To discuss all possible aspects is beyond the scope of this work, but some particular issues for hybrid modeling deserve mentioning. First, in control applications a distinct value needs to be applied and maintaining distributions for repeated sampling is not in the focus. Thus both deterministic and probabilistic methods are reasonable and often perform similarly, because the latter apply a subsequent decision stage to arrive at a well defined output value. Then many of the well-known algorithms internally compute effectively the same type of representation, a “unified regression model” [22] based on a superposition of Gaussian basis functions, as was recently shown in the excellent review [22]. In hybrid modeling, it is more important for the choice of the learning algorithm whether such local learner (e.g., Radial Basis Functions, Gaussian Mixture Models, Gaussian Processes, Locally Weighted Projection Regression, local linear regression) shall be employed, or a learner based on global basis functions like, e.g., Multi-layer Perceptrons or Extreme Learning Machines. The local approach assumes that no extrapolation is needed and desired. Consequently models can be designed such that outputs are zero far away from the training data (e.g., Radial Basis Function network variant described in Section 3.3). This is well suited for trajectory-based approaches, where a particular predefined task has to be tracked. If extrapolation beyond the training data is desired, e.g., in explorative learning, a global internal representation may be better suited. The difference is demonstrated below in Section 3 in the soft-robot use case.
  Second, the data obtained is often sparse and therefore strong biases, that is assumptions about the character of the learning problem, are needed to enable generalization from this sparse data. In practice, it is important to control model complexity in terms of number of basis functions, number of parameters, or by means of regularization as overfitting is often a serious problem. In hybrid modeling applications, some knowledge about the underlying physical processes is typically available. A method to use this knowledge in form of additional constraints for the learner and to mediate the problem of sparse data through such additional bias has been developed in [23] and applied to learn an inverse equilibrium model of the dynamics for the soft robot shown in Figure 1.
  Third, in control applications often the model has to be inverted and/or differentiated and thus a learner that is algebraically differentiable can be very useful. For instance, in inverse velocity control in robotics it is desired to re-compute and invert the Jacobians in every control cycle. Some learners can enable this, as is demonstrated also below in Section 3. Finally, in critical applications it may be desired or required to give guarantees about the learner’s performance. To this aim, in [23] a method has been developed that can proof that after learning certain predefined constraints are observed.
(D) **The performance criterion:** Finally, a performance criterion has to be defined for evaluation of the approach. This is seemingly trivial, but in practice tricky, because the modeling error can hardly be evaluated against ground truth and the performance of the learner on the training data alone is not significant. In most cases, task performance of the hybrid model is more important, which lends in control applications to standard error measures like tracking accuracy along a task trajectory or for a grid of reference positions in kinematic control. However, within a control application often the controller guarantees tracking and more indirect performance criteria must be employed. For instance, the reduction of gains for comparable accuracy has been proposed as criterion in inverse dynamics modeling [14,18], which is also desirable to reduce strain on the mechanism. Unfortunately, this level of performance measurement does not directly feed back to the learning algorithms. Therefore, in most cases the learning system has to be evaluated during training on the data only, independent of the final performance goal. Consequently, special attention has to be paid to the connection between performance criteria with respect to the learning/modeling and task execution.

In summary, hybrid modeling can mediate the problems of both analytical and learning approaches and combine explicit knowledge with a data-driven approach. As compared to other machine learning applications, the context of control and physical plants poses particular constraints and special care must be taken to select the optimal learning algorithm and setting. Contrary to many big data applications, hybrid modeling in this context often has to face small data, requires different methods, and needs a careful choice of the learning biases which reflect respective assumptions about the physical plant. This knowledge is on the other hand also an asset and sets the stage for inclusion of a-priori constraints into the learning system and leads to specialized performance criteria. In the following, we illustrate many of these features with two use-cases that have first been introduced in [15,16], respectively.

## 3. Hybrid Forward Kinematics for a Soft Robot

We first consider the forward kinematics of a soft robot which are particularly difficult to model due to the robot’s flexible structure and material properties (see Figure 1 (left)). The robot’s forward kinematics can be modeled mechanically assuming constant curvature of the segments. This assumption introduces significant errors which are compensated with a data-driven error model in this paper. The inverse kinematics are then solved by inversion of the hybrid forward model. First, the soft robotic platform is introduced. Then, different modeling approaches, including pure analytical and data-driven modeling, are compared to hybrid models. The performance of the hybrid models is finally evaluated for an inverse kinematics tracking task.

### 3.1. Bionic Handling Assistant (BHA)

**Figure 3:**
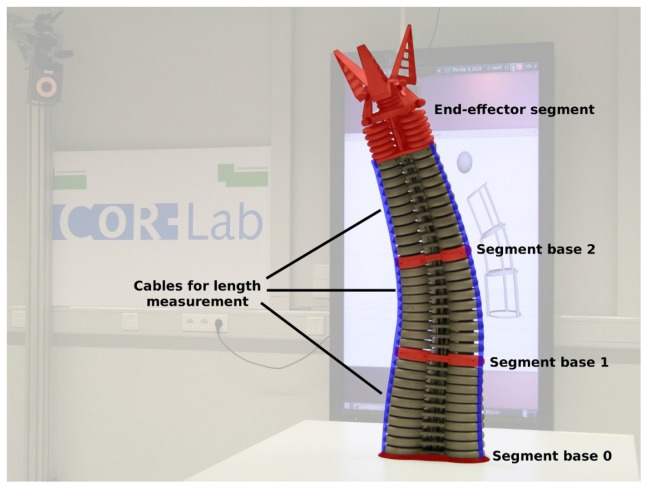
The Bionic Handling Assistant (BHA) comprises three main segments with three air chambers each and a gripper segment. The length expansion of air chambers is measured by cables running along the outer hull of each chamber. Figure reproduced from [19].

The Bionic Handling assistant (BHA, see Figure 1 (left) and Figure 3) has been designed by Festo as a robotic analog to an elephant trunk. The BHA has gathered strong interest because it belongs to a new class of continuum soft and lightweight robots based on additive manufacturing with polyamide [24,25]. The robot trunk is pneumatically actuated and comprises three main segments. Each segment consists of three triangular arranged air chambers. Therefore the main flexibility of the BHA is based on nine air chambers that extend their length in relation to the pressure in those chambers. A fourth end effector segment is also available but was neglected for this work. The robot has no classical, revolute joint angles. Instead each robot segment starts to bend in case that the three chambers reach different lengths. Beside pressure sensors integrated in the air valves, the BHA is equipped with cable potentiometers that allow to measure the outer length of the air chambers providing geometric information about the robot shape. The segments of the BHA together with the attached cable length sensors are depicted in Figure 3.

While the challenge of controlling the segments lengths by supplying the air chambers with appropriate pressures has been addressed in previous work [23,26], this paper focuses on the kinematic control of the end effector. The autonomous exploration of a direct inverse model for controlling the end effector has been accomplished in [7] where the main emphasis was to start model learning from very limited prior knowledge about the plant. In this paper, the idea is to apply the established scheme for solving inverse kinematics for redundant robots by local inversion of the kinematic forward model [27]. We start control design from an approximate model of the robot’s forward kinematics developed in [19] (The constant curvature model is available as open source http://www.cor-lab.org/software-continuum-kinematics-simulation.) and improve this model by learning an error model based on motion capture data.

#### 3.1.1. Constant Curvature Model of the Forward Kinematics

In previous work, a kinematic model of the BHA was developed. The approximate forward kinematic model of the BHA follows a constant curvature approach that combines torus segments in order to represent continuous deformations. Figure 3 shows how the three actuators in each main segment cause a deformation between two rigid segment bases (shown in red). The three measured lengths of these actuators can be used to estimate the coordinate transformation between segment bases, which are then chained in order to get the complete forward kinematics from base to end effector. The only free parameters of the segment model are the segment radii. These radii have been estimated according to a best-fit solution by recording ground-truth data from different movements [19], a typical parameter estimation procedure.

We denote the robot configuration by $q\in R^{9}$, which comprises three length measurements for each segment. The kinematic forward model based on the constant curvature approach is denoted by $x=f^{cnk}\left(q\right)$ (continuum kinematics cnk), where we consider only end effector positions x in this paper for simplicity. This completes the approximate analytical modeling as described in Section 2-(A).

#### 3.1.2. Differential Inverse Kinematics

**Figure 4:**
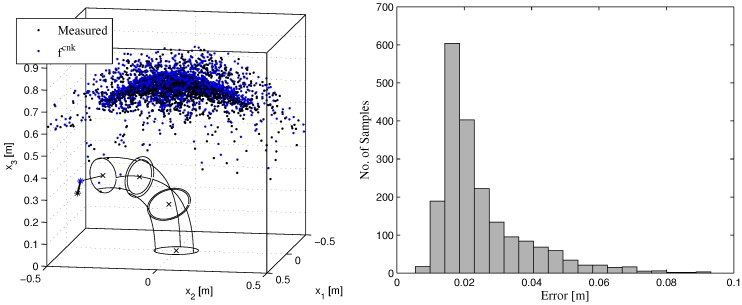
**Left**: Data set with end effector positions measured by a VICON system and estimated end effector positions according to the constant curvature kinematics $f^{cnk}$. The deviation of the kinematic model and the measured end effector positions is shown for an exemplary robot configuration. **Right**: Error histogram for the constant curvature kinematics $f^{cnk}$. Figure reproduced from [15].

The approximate kinematic model allows 3D visualization of the robot (see Figure 1 (left) and Figure 4 (left)) and also inverse kinematic control. To derive an inverse kinematics solver, we consider the differential relation between robot configurations q and end effector positions x:

$$\begin{array}{c} \dot{x}=J\left(q\right)\dot{q},\;\mathrm{where}\;J_{ij}\left(q\right)=\frac{\partial f_{i}\left(q\right)}{\partial q_{j}} \end{array}$$

are the entries of the Jacobian J(q) of the forward model f(q). By inversion of this relation, we obtain the classical differential inverse kinematics control law

$$\begin{array}{c} \dot{q}=J^{†}\left(q\right)\dot{x}, \end{array}$$

where $J^{†}\left(q\right)$ is the Moore-Penrose pseudoinverse of J(q) and $\dot{x}$ is the desired end effector velocity. To accommodate additional constraints into the motion generation for redundant robots, e.g., to avoid joint limits, stay close to a preferred robot configuration and to avoid non-cyclic motions in configuration space, this scheme is typically extended by a term for motion generation in the null-space of the primary task:

(1) $$\begin{array}{c} \dot{q}=J^{†}\left(q\right)\dot{x}+\left(𝟙-J^{†}\left(q\right)J\left(q\right)\right)z \end{array}$$

In this paper, we use $z=\beta (q^{pref}-q)$ to select a redundancy solution close to a preferred robot configuration $q^{pref}$. This completes the description of the control task, which requires inversion of the derivative of the kinematics model and thus poses additional requirements on the hybrid model.

#### 3.1.3. Error of the Constant Curvature Kinematic Model

The assumptions made by the constant curvature model $f^{cnk}$ are violated by the real plant [19,20]. Therefore, the plant kinematics and the kinematic model mismatch. This mismatch and calibration errors [19] result in systematic errors of the forward model and hence reduces the accuracy of the inverse kinematic controller Equation (1). Figure 4 (right) shows a histogram of errors

$$\begin{array}{c} ||x-f^{cnk}\left(q\right)\left|\right| \end{array}$$

between end effector positions x measured by a VICON system [28] with submillimeter accuracy (see background of Figure 1 (left)) and end effector positions estimated by the forward model $f^{cnk}\left(q\right)$ for a set of 2000 configurations q. The histogram in Figure 4 (right) has a heavy tail, which reveals that the error of the forward model is considerable. The mean error is 2.5 cm and the maximal error is 9.3 cm. Note that the accuracy of the inverse kinematic controller (1) is directly affected by the accuracy of the forward kinematic model. Hence, to improve the accuracy of end effector control, it is essential to improve the forward model. This defines the performance criterion according to Section 2-(D).

### 3.2. Data-Driven and Hybrid Forward Models

In order to improve the accuracy of the forward kinematic model, we compare multiple, data-driven and hybrid modeling approaches: Firstly, we consider the pure data-driven modeling that fits a flexible function approximator $f^{dat}\left(q\right)$ to the collected data in order to predict end effector positions x from robot configurations q. While this first approach has to capture the complete non-linearities of the forward kinematics, the second approach restricts learning to the error of the continuum kinematics model $f^{cnk}\left(q\right)$. For the hybrid model

(2) $$\begin{array}{c} f^{hyb}\left(q\right)=f^{cnk}\left(q\right)+\epsilon \left(q\right) \end{array}$$

solely the configuration-dependent error ϵ(q) of the mechanical model $f^{cnk}\left(q\right)$ is learned.

### 3.3. Linear Models, Extreme Learning Machines, and Radial Basis Functions

For learning the completely data-driven model $f^{dat}\left(q\right)$ or the error model ϵ(q), we apply three learning algorithms:

- **Linear Model**: A linear model of the form f(x)=Ax+b is trained by linear regression as a baseline.
- **Extreme Learning Machine** (ELM, [29]): Extreme Learning Machines are feed-forward neural networks with a single hidden layer and an efficient training scheme based on linear regression. The output of the network is computed according to
  (3) $$\begin{array}{c} f\left(x\right)=W^{\mathrm{out}}\sigma \left(W^{\mathrm{inp}}x+b\right), \end{array}$$
  where σ(a)=1/(1+exp(−a)) is a sigmoid activation function applied component-wise to the synaptic summations $a=W^{\mathrm{inp}}x+b$. In comparison to classical training of feed-forward neural networks by gradient descent through error backpropagation, ELMs restrict learning to the connections $W^{\mathrm{out}}\in R^{dim(f(x))\times H}$ from the hidden layer to the outputs. The number of hidden neurons *H* is chosen large in comparison to the number of inputs. The input weights $W^{\mathrm{inp}}\in R^{H\times dim(x)}$ and biases $b\in R^{H}$ are initialized randomly and remain fixed. In this paper, the components of $W^{\mathrm{inp}}$ and b are drawn from a uniform distribution with range [−1,1]. Inputs x are scaled to range [−1,1]. The idea of using feed-forward neural networks with a random hidden layer has been proposed earlier, e.g., in [30].
  Learning aims at minimization of the sum of squared errors with regularization term
  (4) $$\begin{array}{c} \frac{1}{2}\sum_{n=1}^{N}{\left(t_{n}-W^{\mathrm{out}}\sigma \left(W^{\mathrm{inp}}x_{n}+b\right)\right)}^{2}+\frac{\lambda }{2}\left|\right|W^{\mathrm{out}}{\left|\right|}^{2}, \end{array}$$
  where $t_{n}$ is the target output for input $x_{n}$. Minimization of Equation (4) with respect to $W^{\mathrm{out}}$ is a convex optimization problem, which can be solved efficiently by linear regression. The optimal output weight matrix is given by
  (5) $$\begin{array}{c} W^{\mathrm{out}}={\left(H^{T}H+\lambda 𝟙\right)}^{-1}H^{T}T, \end{array}$$
  where H and T collect the hidden neuron activations $\sigma (a_{n})$ and target outputs $t_{n}$ for all n=1,⋯,N samples row-wise. The regularization parameter λ>0 allows for the continuous selection of the model complexity.
- **Radial Basis Functions** (RBF, [31]): We also apply a variant of Radial Basis Functions to learn error models. The basis functions take the form
  (6) $$\begin{array}{c} \sigma_{i}\left(x\right)=exp(-\frac{1}{2\varsigma^{2}}||x-c_{i}{\left|\right|}^{2}), \end{array}$$
  where $c_{i}$ is the center and *ς* the width of the *i*-th basis function, respectively. Training proceeds in two phases. First, the set of basis function centers $\left\{c_{i}\right\}$ is initialized in an unsupervised manner using a simple clustering algorithm. The clustering algorithm adds sample x to the set of basis function centers if $||x-c_{i}\left|\right|>r\;\forall i\in \left\{c_{i}\right\}$. Then, the output matrix is computed according to Equation (5), where the radial basis functions from Equation (6) are used in Equation (3). This RBF implementation has the property that the minimal distance between radial basis function centers and their region of activation can be intuitively parameterized by radius *r* and basis function width *ς*. Further, the basis function responses and hence f(x) approach to zero, i.e., σ(x)≈0, for all inputs x far away from the training data. With this choice of the basis functions, the learner is biased towards learning of error models with heavily restricted extrapolation capabilities. This illustrates the arguments made in Section 2-(C).

#### 3.3.1. Learning of Pure Data-Driven Models and Error Models

The pure data-driven model $f^{dat}\left(q\right)$ is trained with the recorded data ${\left\{\left(q_{n},x_{n}\right)\right\}}_{n=1,\cdots ,N}$ comprising pairs of arm configurations $q_{n}$ (lengths of the air chambers) as inputs and the end effector positions $x_{n}$ measured by the VICON system as target outputs. The hybrid model from Equation (2), in contrast, is trained with pairs ${\left\{\left(q_{n},e_{n}\right)\right\}}_{n=1,\cdots ,N}$ comprising arm configurations $q_{n}$ as inputs and errors $e_{n}=x_{n}-f^{cnk}\left(q_{n}\right)$ of the mechanical forward model $f^{cnk}$ as target outputs. Hence, the learning algorithm identifies the error model ϵ(q) from Equation (2) in the latter case. This implements the data acquisition according to Section 2-(B). Note that here the parameter identification for the analytical model is separated from the learning and uses different data.

#### 3.3.2. Generalization of the Learned Models

We evaluate the accuracy of the data-driven approaches on a set of 2000 configurations q of the robot. To better cover the kinematic effects of each segment and reduce demand for data recording, configurations were sampled randomly per segment first. For each segment, resulting end effector positions have been measured when the three length values for this segment are positioned to 500 random configurations within the admissible length ranges. The remaining segment lengths were kept approximately constant in this scheme. These 1500 data pairs are complemented with 500 additional random configurations for the entire robot, i.e., configurations q are drawn from a nine-dimensional, uniform distribution within the admissible length ranges. The measured end effector positions are shown in Figure 4 (right) for all 2000 samples together with a full random configuration of the robot in simulation.

For evaluation of the generalization ability, the available data is split into a training set comprising all 1500 samples with segment-wise random configurations and 300 samples with completely random configurations. For model selection, 100 samples with completely random robot configurations are used as validation set. The remaining 100 completely random robot configurations are used as test set. Training is repeated five times for the ELMs and RBFs in order to account for random initializations. For ELMs, the number of hidden neurons *H* and the regularization constant *λ* is carefully selected using a grid search. RBFs are trained using r=0.1, which corresponds to a closest distance between two basis functions of 10 cm in configuration space (outer lengths of the air chambers), and ς=0.1. Hence, the basis function responses decline to approximately zero for inputs 20 cm away from the closest training sample. Errors are evaluated according to

$$\begin{array}{c} ||x-f^{□}\left(q\right)\left|\right| \end{array}$$

where $f^{□}$ is either the purely data-driven model $f^{dat}$ or the hybrid model $f^{hyb}$. Here the performance criterion (cf. Section 2-(D)) is the intrinsic error of the learning algorithm as measured in a classical generalization test.

Table 1 shows the average errors for $f^{dat}$ and $f^{hyb}$ when using a Linear Model, Extreme Learning Machine, or Radial Basis Function network for fitting. The results confirm the expectation that it is more difficult to learn the complete forward kinematics in a single model ($f^{dat}$). Because of the non-linear forward kinematics, the Linear Model is not suitable for this task and underfits the data (large training errors in the first row of Table 1. While the ELM can model non-linearities, generalization of $f^{dat}$ then easily suffers from overfitting (large test errors in the second row of Table 1 even though the model complexity has been selected carefully. The hybrid models $f^{hyb}$ in contrast achieve a significant improvement compared to the pure mechanical model $f^{cnk}$ while generalizing well also to novel robot configurations. In fact, a relatively accurate error model ϵ(q) can already be achieved with a model of low complexity, e.g., a Linear Model (cf. third and fourth rows in Table 1. This shows that the inaccurate mechanical forward model significantly reduces the complexity (non-linearity) of learning the error model ϵ(q). However, non-linearities still remain for the error model and the best result is achieved with the non-linear ELM in the hybrid model. The latter result is close to the accuracy that can be achieved with this platform (repeated approaching of configurations is reported to result in approximately 1 cm end effector positioning error in [19]).

The hybrid model with a RBF learner performs comparably well with intuitively selected model complexity (see last row of Table 1). However, it does by design not extrapolate the error model too far from the training data and hence displays a larger maximal test error than the hybrid model with ELM. For some applications, the Radial Basis Function network may nevertheless be preferred due to its interpretability: The basis functions correct errors around regions in the input space where training samples are available. In a well-defined distance to the training samples, the basis function responses decline to zero and the model falls back to its analytical part.

### 3.4. Inverse Kinematics with a Hybrid Forward Model

This section finally shows how to exploit the hybrid forward model with ELM learner to solve inverse kinematics. According to Equation (1), computation of the manipulator Jacobian J(q) of the hybrid forward model $f^{hyb}\left(q\right)$ is required. The entries

$$\begin{array}{c} J_{ij}\left(q\right)=\frac{\partial f_{i}^{hyb}\left(q\right)}{\partial q_{j}}=\frac{\partial f_{i}^{cnk}\left(q\right)}{\partial q_{j}}+\frac{\partial \epsilon_{i}\left(q\right)}{\partial q_{j}}, \end{array}$$

of the Jacobian now comprise a sum of partial derivatives of the mechanical forward model $f^{cnk}\left(q\right)$ and the learned error model ϵ(q). While the derivatives of the mechanical model $f^{cnk}\left(q\right)$ are computed numerically in this work, the partial derivatives of the error model ϵ(q) with ELM learner are computed analytically by

$$\begin{array}{c} \frac{\partial \epsilon_{i}\left(q\right)}{\partial q_{j}}=\sum_{n=1}^{H}w_{in}^{out}w_{nj}^{inp}\sigma_{n}^{\prime }\left(\sum_{k=1}^{dim(q)}w_{nk}^{inp}q_{k}+b_{n}\right), \end{array}$$

where the derivative $\sigma^{\prime }\left(x\right)=\sigma \left(x\right)\left(1-\sigma \left(x\right)\right)$ of the sigmoid function σ(x)=1/(1+exp(−x)) is efficient to compute.

**Figure 5:**
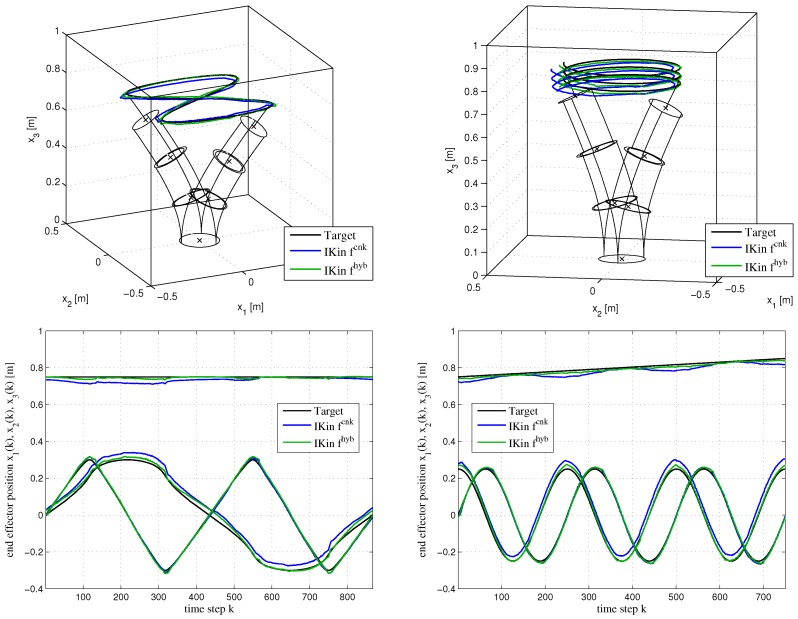
Tracking results for a figure eight motion (**left** column) and spiral motion (**right** column). Target trajectories $x^{*}\left(k\right)$ are shown in black, tracked end effector positions x(k) are recorded with VICON. Tracking results using (1) with continuum kinematics $f^{cnk}$ are shown by blue lines and for the hybrid model $f^{hyb}$ by green lines. The first row shows the trajectory in task space and the bottom row displays the task space trajectories over time steps *k*. Figure reproduced from [15].

**Figure 6:**
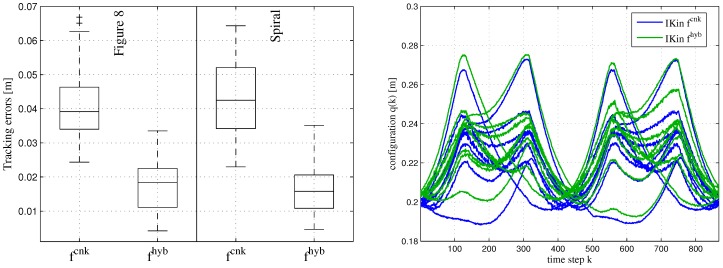
**Left**: Tracking error statistics $\left|\right|x^{*}\left(k\right)-x\left(k\right)\left|\right|$ for both models and trajectories. **Right**: Trajectory in configuration space (lengths of the air chambers) based on the analytical and hybrid model for the figure eight motion over time steps *k*. Figure reproduced from [15].

We demonstrate the inverse kinematic control on the real BHA. We apply the inverse kinematics solver Equation (1) with continuum kinematics $f^{cnk}$ only and with the hybrid model from Equation (2) for two exemplary trajectories (see Figure 5). This implements a performance criterion based on the control task (cf. Section 2-(D)). The hybrid model achieves significantly more accurate tracking results (see Figure 6). The overall average tracking error is reduced from 4.16 cm using only $f^{cnk}$ to 1.67 cm using the hybrid model $f^{hyb}$. The effective change of the trajectory in configuration space (lengths of the air chambers) due to the learned error model is clearly visible in Figure 6 (right). Note that the change of the trajectory in configuration space is not due to a drift along a redundancy manifold during inverse kinematics solving, because we set a preferred configuration $q^{pref}$ (straight robot posture with medium elongation) as null-space constraint in Equation (1). Note also that the trajectories in configuration space with the hybrid model do not display undesired discontinuities during target tracking. Inversion of the hybrid model results in well-behaved and accurate motion generation and the task performance correlates, as expected, with the learning performance.

## 4. Hybrid Inverse Dynamics for a Rigid Robot

### 4.1. The Approximate Analytic Dynamic Model of KUKA LWR IV+

In computed torque control, an inverse dynamics model of a robot manipulator is required to compute torques to track a given task space trajectory with given velocity and acceleration. There are analytical approaches, like the Euler-Lagrange method, the Newton-Euler method and the Lagrange-d’Alembert virtual work principle [32], to calculate the analytical dynamics model if accurate knowledge about the dynamics and kinematic properties of the robotic manipulator is available. However, often the dynamics model cannot be calculated precisely because of non-linearities, friction and temperature effects as well as inaccuracies in the mass information (mass, center of mass, inertia matrix). The latter is the case for the KUKA LWR IV+, the well-known 7-DoF robotic manipulator with lightweight anthropomorphic structure (see Figure 1 (right)) and joint torque sensing, because the manufacturer does not disclose precise model information, which is regarded proprietary company knowledge. We therefore chose a hybrid inverse dynamics model design that is based on so called Independent Joint Learning (IJL, [13]) and demonstrate that the torque error due to unmodeled dynamics can be significantly decreased by IJL for an approximated KUKA LWR IV+ dynamic model.

Formally, the robot dynamics for a *n*-DoF motion with joint angles $q={\left(q_{1},q_{2},\ldots ,q_{n}\right)}^{T}\in R^{n},$ can be expressed in the terms of joint torques $\tau ={\left(\tau_{1},\tau_{2},\ldots ,\tau_{n}\right)}^{T}\in R^{n}$ as:

(7) $$\tau^{\mathrm{real}}=\tau^{\mathrm{ID}}+\varepsilon =M\left(q\right)\ddot{q}+C\left(q,\dot{q}\right)\dot{q}+G\left(q\right)+\varepsilon$$

In Equation (7), $M\left(q\right)\in R^{n\times n}$ is the positive-definite mass matrix, $C\left(q,\dot{q}\right)\in R^{n\times n}$ is the centrifugal and Coriolis coefficient matrix and $G\left(q\right)\in R^{n}$ is the gravitational vector. ***ε*** represents the unmodeled dynamics, that is the error between the true torque obtained from the real robot, $\tau^{\mathrm{real}}$, and the torques obtained from the ID model, $\tau^{\mathrm{ID}}$.

As first proposed in our earlier work [16], we use a respective approximate analytic model derived from a LWR model available in V-REP [33], which provides the full details of every link obtained from CAD data. We implemented this model using the Robotics Toolbox [34] in MATLAB and verified that this model yields essentially the torques also published in [35] by Gaz et al. In [35], the numerical values of dynamic coefficients for this model were estimated through a reverse engineering approach by using a series of link position measurements and data readings in different static configurations. Note that the data used for this parameter identification differs from the data used for learning the error model below. The identified ID model exhibits an error, which is more visible for small joint torques and is most likely due to unmodeled friction in addition to the inevitable inaccuracies in the estimated parameters. This analytic model is our starting point for hybrid modeling in the sense of Section 2-(A).

### 4.2. Data Acquisition

In order to estimate the torque error, we record a dataset consisting of joint angles, velocities, accelerations and torques $\left[q\left(j\right),\dot{q}\left(j\right),\ddot{q}\left(j\right),\tau^{\mathrm{real}}\left(j\right),\tau^{\mathrm{ID}}\left(j\right)\right]$ using a set of excitatory trajectories, where *j* represents the index of the training sample. In an ideal case, the dataset comprises representative samples of all possible joint configurations and trajectories, as discussed in Section 2-(B). However, due to typically high-dimensional state spaces, it is not a-priori clear how to sample this data efficiently. In our case, we consider parametrizations of sinusoidal trajectories

(8) $$q_{i}=A_{i}\cos\left(\frac{n_{i}t}{T}\right),t\in \left[0,T\right],\;i=1,\ldots ,7$$

for this purpose, where we use nine trajectories for training and testing and extend the approach from [35]. We make sure that for comparison purposes the first trajectory is identical to the test trajectory in [35]. For more details on the parameters of $A_{i}$, number of cycles $\left(n_{i}\right)$, and *T* see [16]. The trajectories are generated such that the joint velocities are zero at start and end, with a sampling rate of 100 Hz. Joint velocities and accelerations are computed by differentiating Equation (8). The measured joint torques were filtered through a 4th order zero-phase digital Butterworth filter with a cutoff frequency of 1 Hz as in [35].

### 4.3. Independent Joint Learning (IJL)

In IJL, the torque error $\left(\varepsilon_{i}\right)$ is learned independently for each joint *i* using joint-local information, which is computationally efficient as it relies solely on a joint’s local information instead of the global configuration, but requires to train *i* different learners, each of which is responsible for one joint respectively. We rearrange Equation (7)

(9) $$\varepsilon_{i}=\tau_{i}^{\mathrm{real}}-\tau_{i}^{\mathrm{ID}}$$

to obtain the joint-wise errors as targets for the learner. Obviously, the difficulty of the learning task depends upon the accuracy of the approximate ID model, which may contribute more or less to the torque error.

From the different ways to derive the inverse dynamics model, we know that there are different sets of variables that in principle all completely encode the information that the learner needs. Already Um et al. used in [13] two different input sets $\left(q_{i},{\dot{q}}_{i},{\ddot{q}}_{i},\tau_{i}^{\mathrm{ID}}\right)$ and $\left(\omega_{i},{\dot{\omega }}_{i},\tau_{i+1},f_{i+1}\right)$. Here $\omega_{i},{\dot{\omega }}_{i}$ and $f_{i+1}$ are respectively the angular velocity, angular acceleration and the forces acting on the link *i* in three dimensions. The latter values were calculated using the recursive Newton-Euler method. To implement the recursive Newton-Euler method, parameters like mass, center of gravity, etc., were used from the available model of KUKA LWR IV+ in V-REP as described above. Note that this is a typical feature of the model based approach as discussed in Section 2-(C). The explicit knowledge about different methods to calculate inverse dynamics here provides different options to select suitable inputs. In Section 4.4, we compare the performance of these two sets of inputs and some subsets experimentally and show which input selection can provide the most accurate torque error model. For the actual learning, we again use the ELM learning described in Section 3, although in principle any supervised learning algorithm may be used in the defined setup. This concludes the setup of the learner, cf. Section 2-(C).

### 4.4. Experimental Results

In this section, we discuss the performance of IJL on improving the ID model of KUKA LWR IV+. The performance criterion (cf. Section 2-(D)) is the torque error tested on a part of the recorded ground truth torque data from the robot. Such improvement is assumed to reduce controller gains in the computed torque control approach [18], which was also shown in [14].

The ELMs are employed to learn the torque error with the two different input sets. After performing a grid search over the number of hidden neurons H∈{25,⋯,500} and the regularization parameter $\lambda \in \{10^{-3},\cdots ,10\}$, we selected H=100 and $\lambda =10^{-1}$ as the best parameter configuration for the results described below.

**Figure 7:**
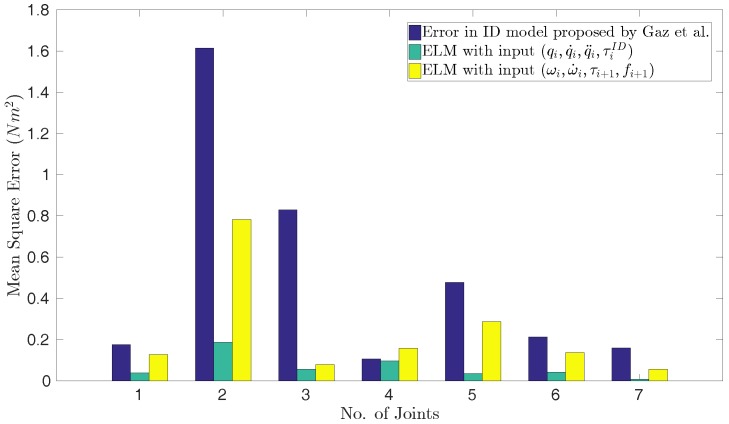
Mean Square Errors for torques per joint obtained by pure analytical and hybrid modeling.

For comparison, we used the same trajectory for testing as in [35] to show the improvement of the ID model, while the other recorded data is used for the training. Figure 7 shows the Mean Square Errors (MSE), where the torque error models are learned by ELMs using the two different input sets. Figure 7 illustrates that although ELMs with input set $\left(\omega_{i},{\dot{\omega }}_{i},\tau_{i+1},f_{i+1}\right)$ improve the ID model, better results are obtained by using ELMs with inputs $\left(q_{i},{\dot{q}}_{i},{\ddot{q}}_{i},\tau_{i}^{\mathrm{ID}}\right)$. This is contrary to the findings by Um et al. [13], which however are based on simulation only. Although $\omega_{i}$ and ${\dot{\omega }}_{i}$ do fully represent the local joint state information, their computation is based on the analytical model and hence is corrupted by the model inaccuracies. The representation of the joint state information by $\omega_{i}$ and ${\dot{\omega }}_{i}$ is furthermore higher dimensional compared to using $q_{i},{\dot{q}}_{i}$ and ${\ddot{q}}_{i}$ only. Thus, an increased demand for training data can be expected in general. We conclude that the incomplete representation of the local joint state information using inputs $\left(q_{i},{\dot{q}}_{i},{\ddot{q}}_{i},\tau_{i}^{\mathrm{ID}}\right)$, which neglects the inter-joint effects along the kinematic chain, is preferable due to its lower dimensionality and independence from the analytical model. However, if the analytical model is already very precise, the input selection by Um et al. may be suitable as well.

**Figure 8:**
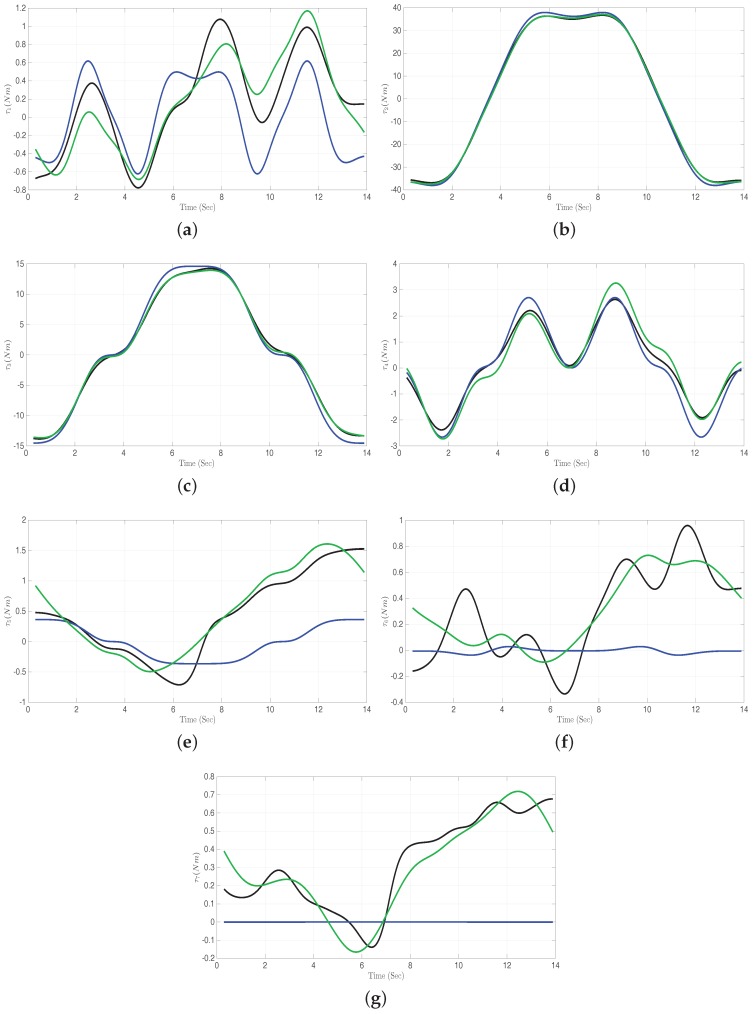
Measured joint torques ($\tau_{i}^{\mathrm{real}}$, black lines), joint torques computed by the analytical ID model ($\tau_{i}^{\mathrm{ID}}$, blue lines), and estimated joint torques ($\tau_{i}^{\mathrm{pre}}$, green lines) from the hybrid model. Results shown for the test data from trajectory-1. (**a**) Joint-1; (**b**) Joint-2; (**c**) Joint-3; (**d**) Joint-4; (**e**) Joint-5; (**f**) Joint-6; (**g**) Joint-7.

In Figure 8, the measured and estimated torques are displayed along the test trajectory. This figure shows a significant improvement of the ID model by means of addition of the error model. This is most apparent for joints five, six and seven, where the analytical model is not capable of capturing the oscillation in the trajectory at all. For the second and third joint, the analytical model fits the overall torque trajectory shape well. However, the absolute torques for these joints are large and hence are the errors in Figure 7. The hybrid modeling approach does improve the torque estimates on the test trajectory for all joints compared to the pure analytical model.

In order to further investigate the input set $\left(q_{i},{\dot{q}}_{i},{\ddot{q}}_{i},\tau_{i}^{\mathrm{ID}}\right)$, different subsets of these input variables are considered and cross-validation for these combinations is performed. Table 2 shows the respective MSEs. The combinations $\left(q,\dot{q},\tau^{\mathrm{ID}}\right)$ and $\left(q,\dot{q},\ddot{q},\tau^{\mathrm{ID}}\right)$ give the best results. Apparently, joint velocities $\dot{q}$ and joint torques from the ID model $\tau^{\mathrm{ID}}$ are the most important inputs to the learner. This supports the assertion that the error of the ID model is mainly due to unmodeled friction, because joint velocities $\dot{q}$ play an important role in friction.

The results show that a significant improvement in the approximate analytical ID model of KUKA LWR IV+ could be achieved. We conclude that the impact of features on error model learning can provide insights into the main sources of analytical model errors, e.g., friction in this example. The hybrid modeling approach can hence also be exploited as tool to test hypotheses about properties of the analytical model.

## 5. Conclusions

While the methods for classical parameter identification and machine or statistical learning approaches have methodological similarities, the main difference is the flexibility of the internal representations used in machine learning. Many of these models are able to fit any function with arbitrary accuracy in theory. However, in practice the selection of a suitable model complexity is the key to achieve accurate generalization to areas of the input space where no data was available for training. This paper describes the methodology for hybrid modeling and shows that the combination of models from a domain, e.g., constant curvature kinematics or inverse dynamics, plus a more flexible machine learning technique that models the error can significantly improve overall accuracy. This additional model learning can be accomplished with data that is typically recorded for parameter identification or model verification or by dedicated training data.

Moreover, we shed some light on the general requirements and properties of the hybrid modeling approach. While the role of the analytical model is mainly to capture the main non-linearities, the choice of the error model needs particular care with respect to model complexity. It is possible to mitigate the black-box character of the error model by choosing learners with intuitively defined regions of activation (e.g., the Radial Basis Function network variant described in Section 3.3). Also, for model exploitation in a control law, the choice of the learner is important with respect to properties like differentiability. A deeper investigation of the impact of the degree of analytical model errors on the hybrid modeling scheme remains an interesting question for future research. How does demand for training data scale with analytical model accuracy? How well do the data-driven approaches cope with regions of high model errors, e.g., locally strong misfits of the analytical model? Which lower bound of the analytical model accuracy indicates the application of a pure data-driven modeling approach? While these are difficult questions and more empirical and theoretical investigations are needed, we believe that the methodology and in particular the machine learning methods are already mature enough for a more widespread application of hybrid modeling along the lines discussed in this paper.

## Figures and Tables

**Table 1:** Average training and test errors together with standard deviations and maximal errors for $f^{dat}$ and $f^{hyb}$ using Linear Models, Extreme Learning Machines, or Radial Basis Functions for fitting.

Model	Learning Algorithm	Training Error (cm)	Test Error (cm)
Data-driven model $f^{dat}$	Linear Model	3.65±3.97(44)	9.8±7.8(43)
Data-driven model $f^{dat}$	ELM (H=375, λ=0.01)	0.44±0.39(3.6)	2.6±1.7(15)
Hybrid model $f^{hyb}$	Linear Model	0.81±0.65(6.1)	1.8±0.8(4.4)
Hybrid model $f^{hyb}$	ELM (H=525, λ=0.5)	0.49±0.46(5.1)	1.3±0.6(3.0)
Hybrid model $f^{hyb}$	Radial Basis Functions	0.45±0.40(3.8)	1.4±0.9(4.5)

**Table 2:** Mean Squared Errors (MSE) of analytical and hybrid inverse dynamic models per joint. For hybrid modeling by IJL, results are shown for different combinations of input variables to the error model on the test set.

Joint	MSE in ID Model [35]	MSE for Predicted Torques after IJL			
		$q,\dot{q},\tau^{\mathrm{ID}}$	$q,\ddot{q},\tau^{\mathrm{ID}}$	$q,\dot{q},\ddot{q}$	$q,\dot{q},\ddot{q},\tau^{\mathrm{ID}}$
1	0.1757	0.0338	0.1541	0.0328	0.0389
2	1.6144	0.1744	0.6258	0.2089	0.1864
3	0.8294	0.0714	0.3385	0.3417	0.0551
4	0.1052	0.0491	0.0607	0.0969	0.0961
5	0.4767	0.0236	0.2734	0.0217	0.0355
6	0.2126	0.0429	0.1022	0.0401	0.0409
7	0.1585	0.0087	0.0487	0.0059	0.0073
